# Transfer of communication teaching skills from university to the clinical workplace – does it happen? A mixed methods study

**DOI:** 10.1186/s12909-021-02834-1

**Published:** 2021-08-17

**Authors:** Jane Ege Møller, Louise Binow Kjaer, Emma Helledie, Lone Folmer Nielsen, Bente Vigh Malling

**Affiliations:** 1 Department of Clinical Medicine, Aarhus University, INCUBA Skejby, Palle Juul- Jensens Boulevard 82, 8200 Aarhus N, Denmark; 2 Centre for Health Sciences Education, Aarhus University, INCUBA Skejby, Palle Juul-Jensens Boulevard 82, 8200 Aarhus N, Denmark; 3 Palliative Care Unit, Department of Oncology, Aarhus University Hospital, Palle Juul-Jensens Boulevard 99, 8200 Aarhus N, Denmark

**Keywords:** Clinical communication skills, Workplace communication teaching, Transfer of teaching skills, Doctor-patient communication

## Abstract

**Background:**

Communication skills learned in the classroom do not transfer easily into clinical practice because they are not reinforced by teachers in the workplace setting and because lack of faculty training restricts the transfer of communication skills in real patient encounters. Trained university-based communication skills teachers often work simultaneously as doctors in clinics. This study explored if and how the skills of these teachers play a role in communication skills training in the clinical workplace.

**Methods:**

We used an exploratory sequential design: a mixed method approach that combined a survey with communication skills teachers, and qualitative individual interviews with these teachers and their educational leaders in clinical departments. The questionnaire was analysed using descriptive statistics. The interviews were analysed using content analysis.

**Results:**

The response rate was 34 %. A majority (93 %) used their communication skills when communicating with patients and relatives. Less than half taught communication in clinical departments. Approximately half of the respondents stated that encouragement from their leaders or colleagues would inspire them to use their teaching skills in the workplace. However, only 20 % had told their leaders about their competencies in teaching communication. One third thought that they needed further teacher training to teach in the clinical workplace.

Qualitative analysis showed that teaching opportunities existed but mainly consisted of random, one-off sessions that came about through the initiative of the communication skills teachers themselves. The teachers described several barriers, such as the challenge of teaching colleagues, as communication relates to identity and hierarchical structures, as well as a lack of requests from colleagues or management, and department culture prioritizing topics relating to medical expertise. None of the educational leaders made use of the teachers’ specific communication skills in a structured way: some saw it as unimportant, while others saw it as a potential resource.

**Conclusion:**

Transfer of the teaching skills of communication skills teachers trained for university-based clinical communication training happened, but to a limited degree. Although both opportunities and barriers for transferring communication skills existed, barriers seemed to dominate, and opportunities for communication skills training in the workplace setting were not used to their full potential.

**Supplementary Information:**

The online version contains supplementary material available at 10.1186/s12909-021-02834-1.

## Background

Communication has been identified as a core clinical skill with major impact on the quality of health care and patient satisfaction [1–5]. Recognising this has led to the development of medical communication skills programs for medical students and residents [6, 7]. Ideally, such training programs include observation and feedback on medical student/resident communication with real patients in the clinical setting; however, this rarely occurs [8, 9]. Available evidence confirms that communication skills taught in the classroom do not transfer easily to clinical practice because they are not modelled or reinforced by teachers in the workplace setting [10–12]. This lack of transfer has been described as a ‘gap’ between what is taught in formal clinical communication skills programs and what occurs in the clinical workplace [13]. Research shows that several factors prevent this transfer. First, communication skills training receives little attention in continuing medical education in general [14, 15]. Second, Brown [16] concluded that transfer of medical student communication skills strongly depends on whether doctors and faculty in clinical practice value the communication skills taught in universities. Third, Perron et al. [17] found that lack of faculty training in the clinical setting is a barrier for transferring communication skills in real patient encounters. In line with this, Rosenbaum [18] argued that it is too often assumed that clinical teachers are effective communicators and /or effective teachers, when in reality most are not.

Thus, there seems to be a tension between the importance attached to communication skills reported in the literature and clinical reality. Most clinical communication research focuses on the transfer of trainee skills, while the transfer of communication teaching skills is explored much more rarely. Perron et al. investigated how communication teaching skills programs could equip clinical supervisors with teaching skills and support communication skills training in the clinical worksplace [19]. Their study showed that the transfer of teaching skills to the workplace was rare and that clinical supervisors’ ability to recognize effective communication skills varied [19]. In another study, Bylund et al. [20] developed an assessment tool for assessing facilitator skills and concluded that training is needed to become a good facilitator of communication skills training. The above studies focused on how to strengthen communication skills of clinical faculty and supervisors in the clinical setting. However, this seems to be challenging.

As suggested by Brown [16], more collaboration between clinical faculty and communication skills teachers would be desirable in order to overcome the transfer gap. University-based communication skills [CS] teachers are trained as teachers in formal clinical communication skills programs and often work simultaneously as doctors in the clinical setting. This group could potentially act as brokers between the formal programs and the clinical workplace and thus contribute to the communication skills training of medical students and residents in the clinical workplace. However, there is a lack of research concerning CS teachers’ experiences of the use of their skills in the clinical workplace. This ‘insider’ perspective could add new and valuable knowledge about the opportunities and barriers for transfer of teacher competencies, and communication skills training in the clinical workplace.

This study explored the following two questions in relation to the transfer of CS teacher competencies:

1. To what extent do trained CS teachers who teach in formal university settings apply their teaching skills formally and informally in the clinical workplace?
2. What are the opportunities and barriers for transfer of CS teacher competencies in the clinical context?

### The setting

In Denmark where this study was conducted, outcome-based education based on the seven CanMED roles was implemented in 2004. Specialist training includes one foundation year and one year in an introductory position before entering specialist training. All postgraduate trainees are appointed an educational supervisor in each rotation. Furthermore, in all departments with specialist training, an educational leader (EL) is appointed among the consultants.

Denmark is generally aligned with the international tendency to increase clinical communication skills training. All Danish medical schools now have communication skills programs [21]. Several continuing medical education initiatives have been implemented [15, 22], and as one of the only countries in the world, Denmark has, since 2004, a mandatory communication skills course for all first year residents.

An example of a university communication teaching skills program is the course at Masters level at the Medical School of Aarhus University. Approximately 134 medical doctors have been trained to teach the courses in this program during the period 2009–2019. These doctors have attended seminars and workshops tailored to the course and gained skills in teaching communication, including facilitation of peer feedback, peer supervision, small group teaching, role play and video supervision [21]. See Additional file 1 for a description of the communication courses and teacher training. Alongside teaching communication, the doctors perform clinical work in hospital departments and general practice. This would seem an ideal opportunity for both teachers and departments to use communication teacher skills to ensure effective patient communication and improve the feedback culture in the department.

## Methods

**Fig. 1:**
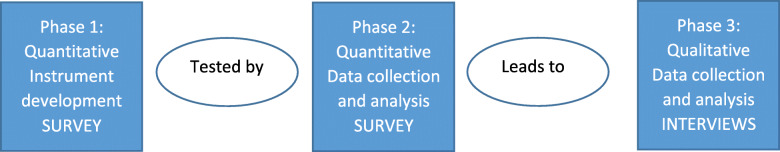
Research design

In accordance with our research question, the study had an exploratory sequential design with two distinct phases: a quantitative phase employing a questionnaire, followed by a qualitative phase employing interviews [23]. The purpose of the quantitative phase was to give an overview of the extent to which teacher skills were used or not used, as well as insight into the opportunities and barriers for using teacher skills in the workplace. The purpose of the qualitative phase was to provide in-depth explanations of the findings from the quantitative data analysis. Data triangulation was used to increase the validity of the study [24] and to provide a rich picture of the phenomenon being studied: communication skills teachers’ use of their skills in clinical practice [25] (Fig. 1).

### Participants

Communication skills teachers who (1) either taught or previously had taught medical students at Aarhus University and (2) were employed as residents/doctors in the Central Denmark Region were included in the study. A fixed population of 134 CS teachers was identified through university records; they received information about the project and an invitation to answer the questionnaire via email. Invitations were sent by the course manager, whom all of the CS teachers knew. Reminder emails were sent to those who did not respond within the first three weeks.

Based on questionnaire data analysis, participants were divided into sub-groups: (1) those who used their teaching skills in their past jobs or (2) present job, and (3) teachers who did not use their teaching skills in their past jobs or (4) present job. Participants from each sub-group were selected for interviews to explore relevant themes based on the survey analysis. The sample strategy for interview participants was to maximize the range, as described by Weiss [26]. We also aimed for variation in terms of gender and specialty. A total of five CS teachers were selected for qualitative interviews. They were between their 2nd and 4th year of specialist training in neurosurgery, oncology, general practice or gynecology. Two were male and three female. Furthermore, two educational leaders (EL) were invited for supplementary interviews to provide a second perspective on opportunities and barriers for using teaching skills at the workplace. Here, the criteria for selection were: (1) that they were consultants from departments where CS teachers had been employed (gynecology and oncology), and (2) that they were available for an interview. The participants in the qualitative study were invited by email from the first author and informed consent was obtained from all by email. We informed the CS teachers that educational leaders would be invited to participate in an interview, assuring anonymity.

### Data collection

The questionnaire was designed by the authors through a modified seven-step approach described by Artino et al. [27]. First, the authors developed a list of questions, based on relevant literature. Then the relevance of questions was discussed by a focus group consisting of three experienced CS teachers (expert validation). Finally, the questionnaire was pilot-tested by 10 CS teachers at the Southern University in Denmark. The pilot test resulted in minor changes in the wording of two questions, and the final questionnaire (included in Additional file 2) comprised 18 questions. The questionnaire was divided into seven themes (see Table 1). Some questions were expanded with additional supplementary questions, depending on the respondents’ answers. (E.g. If the respondent answered “Yes” to the question: “Are you still an active teacher on a communication course?”, a drop-down menu popped up giving the respondent the opportunity to mark one or several of various communication courses mentioned). Answering options in the questionnaire were either a graduated specified scale (e.g. For the question: “Who did you teach?”, the various possible responses were specified: 1) the whole department, 2) the whole group of physicians, 3) only physicians with lower education level than yourself, 4) only medical students etc.), a seven-point Likert scale (range: 1 = not at all; 7 = to a great extent) or free text (only one question). The questionnaire is provided in Additional file 2.

**Table 1:** Questionnaire themes

Questionnaire themes	
1	Reasons for teaching communication skills at the university
2	Experience as teacher
3	Competences as teacher
4	Challenges with communication skills teaching outside the university
5	Kinds of teaching outside the university
6	Colleagues’ interest in teachers’ skills
7	Demographic data

Based on discussions of the results from the quantitative analysis, the authors developed an interview guide (Additional file 3) for the qualitative interviews. The purpose was to further explore how SC teachers used their skills in the workplace and identify opportunities and barriers relating to the use of CS teacher competencies. Interviews were conducted as phone interviews by the first author about one month after the questionnaire. The interviews were audio-recorded and transcribed verbatim.

### Data analysis

The questionnaire data were analyzed descriptively and key findings were identified by the authors. The interviews were analysed using thematic analysis [28]. The first author made an initial thematic coding which was discussed and adjusted by all of the authors. The second author then combined themes with key findings from the survey in order to reach a full picture of both convergence and inconsistency in the data material. Finally, all authors discussed these findings and made final alterations to the themes of the qualitative material. Disagreements about codes and themes were resolved by revisiting the transcriptions until consensus was reached.

### Ethics

The study was approved by the Danish Data Protection Agency (Journal number 2016-051-000001, 1780). The study did not require approval from the Central Denmark Region Committee on Health Research Ethics, according to the Consolidation Act on Research Ethics Review of Health Research Projects.

## Results

In the following, we first present the results from the survey, and then we explore these in more depth through the results from the qualitative analysis.

### Questionnaire

The response rate was 34 % (45 of the 134 identified communication skills teachers). Most respondents were female (80 %) compared to a female/male ratio of 60/40 in the uptake of medical students [2]. Most respondents were under specialist training (75 %) or already specialists (16 %). Teachers were mainly specialists in or aiming for general practice (42 %), oncology (13 %) or psychiatry (7 %). Almost all chose to teach communication skills because they found it essential for medical practice (91 %), and well over half (62 %) because they wanted to become better communicators themselves.

Most teachers taught more than one module (see Additional file 1) and for more than one year. Approximately two thirds were still teaching at the university. 75 % of the respondents characterized themselves as experienced or very experienced communication skills teachers.a

The main finding concerning communication teachers’ use of their skills was that 93 % reported using communication skills in their own communication with patients and relatives, while less than half of the respondents (approximately 40 %) used their communication teaching skills to teach in clinical departments (see Table 2). Three main reasons to refrain from teaching were mentioned by the respondents: (1) it had not occurred to them to offer teaching communication skills, (2) they found it difficult teaching older colleagues, and (3) medical and clinical issues seemed to have higher priority in the clinical setting.

**Table 2:** Shows the percentage of communication skills teachers who use their teaching skills in the clinical setting (past and present) and the format used when teaching communication skills in the clinic

	Use of teaching skills	
	In present job	In former job(s)
Percentage (N)	38% (17)	42% (19)
Teaching formats		
Planned sessions	47% (8)	21% (4)
Repeated sessions	35% (6)	0% (0)
Single presentation	24% (4)	74% (15)
Spontaneous (bedside)	76% (13)	63% (12)

When asked what it would take for them to teach in the clinical workplace, approximately half of the respondents answered that they would have to be encouraged to teach by their leaders or colleagues. However, very few (20 %) had told their leaders about their competencies in teaching communication. They also reported that a change in culture towards greater acceptance of communication skills training in the clinical workplace was necessary before they would consider teaching colleagues. One third thought that they needed further teacher training to teach in the clinical workplace.

Table 2 shows the number and percentage of university communication skills teachers who use their teacher competencies to teach communication in their clinical position and the kind of teaching they deliver in the clinical setting.

### Qualitative analysis

In the following analysis, we first present the results from the CS teacher interviews, and then the results from the interviews with educational leaders.

### CS teacher perspective

The analysis of the CS teachers’ perspectives showed three main themes, namely (1) experiences with university teaching, (2) opportunities for workplace teaching, and (3) barriers to workplace teaching. These will be presented and elaborated on below.

#### Experiences with university teaching

All CS teachers found it highly rewarding to teach CS to medical students at the university and ‘pass something on’ to the next generation of doctors. They explained how they used the skills they had acquired through teaching in their own patient encounters, as expressed in these quotations:

*“I use it every day - for example, when I work in the out-patient clinic” (G, female).*

*“The more you teach communication, the more nuanced you become yourself, because all of the basic skills are repeated and reinforced”. (B, female)*

#### Opportunities for workplace teaching

CS teachers described that CS teaching in the workplace was mostly random, informal bedside teaching of junior colleagues /students. Some CS teachers primarily used their teaching competencies informally when providing a colleague with feedback. Here, they often chose to use the interview techniques and strategies from the in-depth interview style which they knew from teaching at the university. This technique was seen as helpful for providing feedback and dealing with colleagues who had experienced something difficult.

*“And sometimes – if I am senior, I would do a feedback session, which I would pretty much structure like an in-depth interview. Also, when giving feedback to students in general, for example, I have asked: ‘What would you like feedback on now?’ and ‘What do you think you can learn from the next patient?’ – that kind of thing. It has been useful, but mostly done one-to-one.” (M, male)*.

This only took place when the CS teacher was senior or peer to the person receiving feedback. In addition, the initiative to use these skills came intuitively and unplanned from the individual CS teacher, as opposed to being part of department guidelines. The skills primarily related to the process of providing feedback, not the communication skills used in patient encounters.

### Formal teaching

Formal teaching occurred more rarely, and when it did, the CS teachers described that they occasionally took the opportunity to fill out short teaching sessions with communication content. This was often linked to mandatory teaching sessions in the departments where young doctors were expected to teach about an individually chosen topic on one or more occasions, e.g. a five minute presentation after the morning conference. Some chose communication as the topic, as witnessed in the following quotation:

*“And once, I taught communication to all the doctors in the [name of department], because one morning they didn’t know [who to give a short presentation]. So I thought, ‘I might as well tell them about the communication I teach’.” (M, male)*.

The most comprehensive example was described by a CS teacher who taught about feedback at a 3-hour meeting for a team of residents/young doctors and nurses, as shown in the following quotation:

*“I used it at the psychiatric department where I taught feedback at a meeting. And people were happy about it. It was really only that you had an optional theme you could choose and I chose collegial supervision as the theme and taught a bit about communication and giving feedback to colleagues. I chose that because I had witnessed some situations that weren’t ideal. So, I thought there was something my colleagues could learn. A lot of them said afterwards that they had used it and it was really good. However, there wasn’t any follow up. It was a one-off.”(B, female)*.

All opportunities for formal teaching originated from the described individual choice of communication as the topic for the mandatory education in the department, as opposed to colleagues or management, for example, requesting it. As seen in the quotation above, several described that their reason for choosing communication skills (as opposed to other more medical, expert-related topics) was witnessing colleagues who communicated in an inadequate or undesirable manner, who were therefore in need of communication skills.

Another aspect was how colleagues received the teaching. This was described as ranging from very positive, as in the above quotation, to more mixed as reflected in this CS teacher’s experience:

*“And I honestly can’t remember if it was after a nightshift or when it was… But the teaching I did was quite basic about rephrasing, open and closed questions, meta-communication that kind of thing – and empathy – all the things I taught 7th semester university students. My thought was… there were a lot of consultants present, and even though on the surface they seemed to think – that was what I thought from their expressions – that it was a bit too trivial, I’ve also seen them communicate with patients and not everyone is really that good at it. So, I thought that they could learn a thing or two. They were not outright condescending; they were nice and friendly, but might just turn their noses up a bit. But I had a good feeling, because I felt I had helped a couple of them with a brush up of the simple rules, because not all are following them, I have to say. And they said, fine and thank you, but no one came and admitted, ‘We actually needed that teaching in communication’ or ‘I could really use that’. It was a bit disappointing. (M, male)*

#### Barriers to teaching

The CS teachers described barriers that related to four subthemes: (1) the challenge of teaching colleagues as communication relates to identity, (2) hierarchical structures, (3) no request from colleagues or management, and (4) department culture and the apparent prioritizing of topics relating to medical expertise. These will be presented below.

### The challenge of teaching colleagues as communication relates to identity

Many described how communication was perceived as relating to people’s identity, and therefore a skill that was more challenging to teach. Several expressed that this related both to personal identity and/or professional identity as a doctor:

*“The thought has crossed my mind [to teach communication skills in the workplace]: ‘Well, that might be an option’, also as I have experienced that others haven’t got these skills. But then I thought: 1) ‘They probably won’t think it’s that bloody interesting to hear about’ and 2) ‘Will I step on someone’s toes?’ Would they somehow feel that it was too lecturing (…) because my feeling is that communication has to do with how you are as a doctor, as opposed to the hard medical expert stuff.” (L, male)*.

Communication was thus understood as another kind of skill than the ‘medical expert stuff’, and constructive criticism was seen not as much as correcting a skill as criticizing someone’s personality.

### Hierarchical structures

Hierarchy also played a significant role, and was considered a barrier by the CS teachers. They described teaching seniors as challenging. More specifically, they indicated that more senior colleagues might have difficulty in taking CS teaching in:

*“Some senior colleagues really need it, but I’m just afraid to offend someone. Old dogs find it hard to learn new tricks. And this might be something that I’m imputing them, but I feel uncomfortable coming along and telling experienced GPs or consultants with 20 years of experience that it would be more appropriate to talk to their patients “my” way.” (L, male)*.

### No request from colleagues or management

Some expressed that they hoped to gain recognition from senior colleagues by teaching in the departments, and that they anticipated that teaching on medical expert themes resulted in this to a higher degree than teaching communication:

*“Well, I also think it has something to do with wanting recognition, and I feel I have gained more recognition for teaching about some rare disease [as opposed to CS], where senior doctors think, “Wow, that was exciting. This is the right thing, here I have something to say”, because they had expert knowledge about that disease. (M, male)*

### Department culture and the apparent prioritizing of topics relating to medical expertise

The CS teachers also mentioned barriers that related to department culture and the prioritization of certain topics. In their experience, more medical expert-oriented themes were prioritized in the time set aside to teach. Sometimes, this was seen as necessary due to time constraints, but at other times it was seen as a discrepancy between departmental policy and reality:

*“The official statement is that they attach importance to communication, for example, in job interviews. But I have never experienced someone actually saying, ‘I can see that you teach communication skills - would you give a presentation about it?’” (L, male)*.

Some even described that the culture of many departments was a barrier and this type of training would not be valued by colleagues.

*“I would say that it’s because the culture is not like that. It’s both the culture and that people are busy. You could choose to talk to the EL and then teach communication, but then again what is relevant? Because most would think “Communication… we know how to do that, and then they would walk out - you know, pretty typical.” (D, female)*.

This varied from department to department where some were considered more positive than others.

All explained that support from management and colleagues would help them feel that teaching CS was legitimate. Requests from management or senior colleagues would make a difference, and would make it possible for them to bring their teaching skills more into play.

*“If I had the support of people higher in the hierarchy than me that backed this up, then absolutely.” (C, female)*.

While management support was seen as key to providing a legitimate position to speak from, it was not considered enough for this type of teaching to be successful. Senior staff members’ views were deemed equally important and these were described as varying across departments, as witnessed in the following quotation:

*“In neurosurgery, they probably wouldn’t mind because communication is taken relatively seriously. But in the orthopedics department, well (laughs)…I might as well say it as it is. They would just laugh at it and say yes, we know how to communicate and they would just walk out. Some would stay and listen, but it wouldn’t necessarily be something they would use.” (G, female)*.

They all expressed that more training and teaching in communication would be a good idea if they had the support described.

### Perspective of the educational leaders

Questions for the educational leaders were about the importance of communication skills in the department, and knowledge about as well as use of employee communication teacher skills. Two themes emerged from the interviews: (1) CS teacher competencies are ‘not needed’, and (2) CS teacher competencies are ‘a potential resource’.

#### CS teacher competencies are ‘not needed’

CS teachers’ specific competencies were perceived as unimportant in the department. This is witnessed in the following quotation where an EL reflects on what she would think if she heard of any doctor in the department being a CS teacher at the university:

*“Well, I would think that was fine, but I would also think that I really don’t need to know. We already prioritize communication; I think that has to do with our specialty.” (A, female)*.

It was described that communication skills were assessed in the department using the mini-clinical examination method:

*“In our department, we actully often let the nurses assess all the soft skills including communication [in mini-CEX assessments], and then the doctors assess the more hardcore medical expert facts. Because the nurses work closer with them [trainees]. (A, female)*

As observed, communication skills were here described as ‘soft skills’ which the nurses assessed, as opposed to the doctors supervising ‘the hardcore facts’. While no formal CS teaching in the department had been taking place, it was regarded as allowed, but unnecessary. This is seen in the following quotation:

”*I would say ‘Feel free’. Everything is allowed. If you want to talk about that [communication skills] in your five minute presentation in the morning that would be fine. I’ve just never seen it. (A, female)*

#### CS teacher competencies are ‘a potential resource’

Another perspective was when the ELs saw these competencies as an unused and potential resource, as witnessed in the following quote:

*“I don’t think that we make use of it, and I think that’s too bad. I actually think you could make much better use of their competencies (…) because they know stuff that we, their colleagues, could learn from. They didn’t need to keep quiet about that. I wish they would be more open about it.” (B, female)*.

More specifically, it was mentioned that the CS teachers could play a role in improving the supervision of colleagues, teaching and in general being seen as ‘super-users’ in communication more generally. The ELs stated that knowing from the beginning that a newly employed doctor had CS teaching competencies would enable her to consider how these competencies could come into play. However, it depended on the CS teachers actively taking the initiative to inform about their skills.

## Discussion

This study showed that teachers only applied their teaching skills and competencies in the clinical workplace to a limited degree. Opportunities for teaching were mainly informal, random bedside teaching or one-off sessions prompted by the individual CS teacher and not the hospital departments. Although both opportunities and barriers for transferring communication teaching skills existed, barriers seemed to dominate in relation to formal teaching opportunities. Several factors influenced this: the individual characteristics of the CS teachers, the design of the training, and the working environment. This resonates with the theoretical framework for factors influencing transfer of training described by Grossman & Salas [29], which will be discussed in the following.

### Individual characteristics of the CS teachers

Individual factors enabling transfer [29] were observed in our study. For example, a majority of the CS teachers perceived communication skills as highly important, and were highly motivated ‘to pass something on’ to medical students. However, this was contradicted by the low formal use of CS teacher competencies in the workplace. Available evidence has concluded that physicians who received CS teacher training had personal benefits in terms of their own *communication* skills, even though the transfer of new *teaching* skills to the workplace was limited [19]. This supports our findings and indicates that barriers exist both inside and outside the individual teacher.

An individual barrier seemed to be that communication was considered a particular kind of skill relating to identity. According to the CS teachers, this made it more difficult to teach to colleagues than medical expert topics, especially if they were senior colleagues. This is congruent with a study which found that fear of appearing to criticize a colleague’s personality when providing feedback was a challenge for workplace-based communication skills training and collegial feedback [15]. Our results add new insights to this by showing that several CS teachers did observe the need for improving the communication skills of both junior and senior colleagues. However, the CS teachers were hesitant to act on this observation without management support.

It is remarkable that half of the participants stated that management support and encouragement would increase their use of CS teacher skills in the clinical setting, while only 20 % actually told their educational leaders about their competencies. A possible explanation for this could be that CS teachers primarily show “accepted” parts of their professional identity, such as medical expert knowledge and skills when they are at the workplace. In the clinical context, shared professional identity is defined by tacit rules that must be followed in order to obtain acceptance as “one of us” [30]. If CS teacher skills are not acknowledged as important in the workplace, it will be more difficult to use them. This is different in the university CS teaching courses, where the core skill is CS teaching. This explanation is supported by Kold [31], who described how a person’s identity has several sides which become visible in different contexts.

### Training design

An authentic learning environment is another factor described as important for transfer [29]. In our study, the difference between the university context and the clinical setting might be one explanation as to why some CS teachers never considered teaching in the workplace. Furthermore, one third of the CS teachers expressed the need for further training that would enable them to tailor CS training to specific clinical contexts. This aligns with the fact that CS teacher training of participants in this study only focused on university teaching, not on the use of CS skills in the workplace. Other studies have shown that training in the clinical classroom, from a trainee perspective, differs from the more chaotic reality of the workplace [18], and recommended that workplace-based communication teaching should move beyond uniform, basic skills, and adapt to the clinical context [32–36]. Our results highlight the importance of faculty development related to authentic learning environments for CS teacher skills to be used in the clinical workplace.

### Work environment

Most of the barriers to transferring the CS teachers’ skills were found in the working environment and related to factors such as transfer climate, opportunity to perform and support from superiors [29]. One barrier related to the role of hierarchy. Most of the CS teachers were early in their career, which probably made it more challenging for them to teach communication skills to their seniors, despite the fact that they had the expertise and noticed that seniors lacked these skills. With inspiration from Lave and Wenger’s concept of legitimate peripheral participation [37], one may understand that this paradoxical pattern emerges because CS teachers are ‘marginal participants’ in the community of practice, even though they hold expert knowledge that other more central and senior community members do not.

Overall, few opportunities were available in the workplace. The CS teachers had to take the initiative themselves, which made it difficult as other medical expert topics were perceived as more prioritized and recognized as more important in formal teaching. This is in accordance with other studies that showed that medical expert knowledge seems to be more important than communication skills in the workplace [38–40]. We even found that some educational leaders viewed CS teachers’ skills as unimportant for practice, which adds to this picture.

Some CS teachers described this as a ‘culture’ of not valuing communication or a ‘discrepancy’ between official statements about the importance of communication skills and clinical reality. This was exemplified by the department where “hardcore medical expert facts” were taught by doctors, and communication “soft skills” by nurses. Other studies support this finding, showing how workplace institutions attribute little value to communication skills and training [41, 42]. However, the culture in departments was described as varying tremendously. In some departments, communication skills were highly valued, while in others they were not, which was also witnessed in the different perspectives of the educational leaders. This variation can be seen as a barrier as early career medical work involves many shifts between departments.

Finally, the evidence-based or evidence-informed practice so deeply integrated in healthcare professions obviously does not (yet) include skills like communication. Even if the importance of skilled patient communication is widely acknowledged, teaching communication in the workplace is not at all close to reaching its potential. This study thus points at a hitherto unexploited resource regarding improvement in patient communication. Other studies recommend enhancing faculty training in workplace-based communication skills teaching and feedback [43, 44]. This is congruent with a more general tendency in medical education, where faculty development and ‘teaching teachers how to teach’ has been acknowledged as a crucial asset in medical schools [45]. Our study, however, shows that more training is not enough in itself, as doctors who are already trained CS teachers and have adequate teaching competencies are not using their skills in clinical departments. This may be because practitioners do not recognize the need for improved communication, or because other topics are regarded as more important. It may otherwise be that an implementation strategy is absent or the conceptual frameworks are too foreign for the staff. Thus, this study has revealed many of the barriers for putting evidence into practice as described by Nutley et al. [46]. At the same time, it should be recognized that changing practice or culture in hierarchical professional organizations is by no means an easy task [47, 48] – especially not for new-comers in a department.

Various solutions to the knowledge-doing gap exposed here could be proposed, although they can only be speculative since they were not part of the investigation: CS teachers should be spotted and requested to use their skills in the department by their leaders. Various models for communication skills training in the clinical setting should be tried out, since the same model might not fit into the cultures, values and assumptions in various departments [47]. The widespread use of videos in both pre- and postgraduate communication skills training might be used to a greater extent in departments to make communication skills training a mutual project in the department [15]. There is a need for further studies regarding communication skills in the workplace.

Our study has limitations. We based our study on data from surveys and interviews and thus explored peoples’ perceptions of their educational practice, and not necessarily the practice itself. Observation studies would have enabled us to gain insights into how workplace teaching actually takes place, and would have been an interesting dimension. Still, we find that our combination of quantitative and qualitative data, and our inclusion of the perspectives of the CS teachers and ELs has provided a rich picture.

Our response rate of 34 % is low and is therefore a limitation. It should however, be seen in relation to investigating a fixed population (134), which strengthens our results. In addition, our use of qualitative interviews also served to triangulate and validate the findings of the questionnaire. We found repeated themes and variations, but we cannot rule out the possibility that new themes would have appeared had we conducted more interviews. We did not include member checking to secure validity, which might also be seen as a limitation. Furthermore, the number of interviews (seven in total) could also be seen as a limitation. However, qualitative research recommendations support that few interviews can provide very rich material on a narrow subject when analysed in depth [49]. All authors coded the material, thus employing researcher triangulation to validate the findings, and the mixed methods approach contributed with data triangulation. These methods increase the credibility of the findings. We also found an over-representation of female CS teachers in our material when compared to the gender distribution of doctors. This may be an expression of a general overrepresentation of female CS teachers. Another limitation is that this study took place in the Danish medical education system with its local contexts and specific characteristics. Therefore, our findings might not be transferrable to other contexts.

## Conclusions

Transferral of CS teachers’ teaching skills trained for university-based clinical communication training happened, but only to a limited degree. Opportunities for teaching existed but were mainly random and one-off sessions, prompted by the individual initiative of the CS teacher, and not requested by the hospital departments. CS teachers described several barriers including the challenge of teaching colleagues as communication relates to identity, hierarchical structures, lack of requests from colleagues or management, and a department culture that prioritized medical expert topics. None of the educational leaders had made use of the CS teachers’ specific skills in a structured way; some saw it as unimportant, while others saw it as a potential resource. Thus, although both opportunities and barriers for transferring CS skills existed, barriers seemed to dominate, and opportunities for CS training in the workplace setting were not used to their full potential. Further research is needed to show how the skills of CS teachers can be utilized in clinical departments.

## Supplementary Information

**Additional file 1.** Description of the communication courses and teacher training.

**Additional file 2.** Questionnaire.

**Additional file 3.** Interview guides, qualitative study.

## Data Availability

The data sets generated and analysed during the current study are not publicly available, primarily due to discretion and to ensure the anonymity of the participants, and secondarily because all data sets are in Danish. The data set from the questionnaire are available from the corresponding author on reasonable request. Data from the interviews cannot be shared since the participants were promised anonymity and because of the small number of interviewees.

## Abbreviations

CS teachers: Communication skills teachers
EL: Educational leader
