# Supplementary material for: Transfer of communication teaching skills from university to the clinical workplace – does it happen? A mixed methods study
Source: BMC Med Educ. 2021 Aug 17;21:433. doi: 10.1186/s12909-021-02834-1 (PMC8369612; doi:10.1186/s12909-021-02834-1)
Supplement: Supplementary file 1 — Additional file 1. Description of the communication courses and teacher training. [file 12909_2021_2834_MOESM1_ESM.docx]

# **Faculty development program in communication skills teaching of medical Masters students at Aarhus University, Denmark**

## Pre-graduate communication skills courses:

The communication skills training at the Masters (MA) program in Medicine at Aarhus University, Denmark, consists of 4 modules progressing from MA year 1 to MA year 3 (the MA program lasts 3 years in total). Module 1 involves communication skills training with actors and role-play. Module 2 involves peer supervision on the student’s own cases concerning communicative challenges experienced in clinical placements. Module 3 involves video supervision of the student’s own video-recorded patient encounter. The last module is module 5, which involves peer supervision like Module 2^[[1]](#footnote-1)^.

The modules are structured as small-group teaching, where teaching-learning activities systematically include short presentations, different interactive case-scenarios, and different feedback and peer supervision methods.

The facilitators and supervisors in these pre-graduate communication modules are primarily physicians. All teachers must complete the relevant faculty development course which consists of:

1. A course for module 1 and 3 facilitators, including a 3-hour introduction to the module content and teaching-learning activities, plus co-facilitation of two or more modules with an experienced facilitator who gives a mandatory evaluation of the new facilitator to the course coordinator.
2. A course for module 2 and 5 supervisors, including a 3-day course that introduces the module content and training in the peer supervision methods used in the modules, plus participation in a mandatory mid-term supervision meeting.

**Methods:** The facilitator and supervisor training are based on two methods:

1. The courses for modules 1 and 3 are inspired by the apprenticeship approach, meaning that the facilitators primarily learn by observing and working together with an experienced facilitator.
2. The courses for modules 2 and 5 are based on principles of professional peer supervision, meaning that the supervisors primarily learn together by gaining insights into and experience of the same exercises they will be training with the students.

Both courses include reflective exercises related to the facilitator and supervisor role.

1. Module 4 is not described here because it is not part of the faculty facilitator and supervisor training, as it has a specialised teacher program about communication with psychiatric patients. [↑](#footnote-ref-1)
