# Supplementary material for: Transfer of communication teaching skills from university to the clinical workplace – does it happen? A mixed methods study
Source: BMC Med Educ. 2021 Aug 17;21:433. doi: 10.1186/s12909-021-02834-1 (PMC8369612; doi:10.1186/s12909-021-02834-1)
Supplement: Supplementary file 3 — Additional file 3. Interview guides, qualitative study. [file 12909_2021_2834_MOESM3_ESM.docx]

**Appendix 3. Interview guides, qualitative study**

**Interview guide: Educational Leaders**

- Would you say communication is important in your department?
- Are communication skills taught? How – formally/ad hoc?
- What other topics are taught in the department?
- Are you aware of any of your employees having CS teaching competencies?
- Do they bring to your attention their potential use, do they offer to teach?
- What opportunities for teaching exist?
- Is it something that is requested among doctors in the department?
- Would CS teaching cause challenges?
- If you knew about CS teachers, would it be useful to bring CS teaching skills to play in the department?
- What would it take for you to use them?
- What do you think are the reasons for you not using use them?
- Do you remember CS skills being used in any training or competency assessment?
- Is it relevant for the department at all?

**Interview guide: CS teachers**

**Personal experience with university teaching**

- Which university semesters did you teach CS?
- What do you think about teaching?
- Was it meaningful to you - how / how not?
- How do you estimate your competency as a teacher?
- What were you good at?
- What was challenging?

**Communication teaching skills in clinical practice**

- Do you think communication skills training is relevant in your specialty / department? Why/ why not?

**Reasons for not using CS teaching skills in former or current employment**

- Was there focus on communication in the department you worked at? How was this evident? Was communication considered important in the department?
- Looking back, why do you think you did not use your teaching skills more actively?
- What do you think about not bringing your teaching skills into play?
- What would it take for you to do so?
- What other topics did you teach at the department?
- Why did you choose xxx instead of communication?

**Department culture**

- What characterizes the educational culture?
- What is the learning / education culture like in your department?
- What is the working environment like, in general?
- Which of the seven roles of the doctor are in focus?
- Is there a focus on communication skills in your department?
- Why do you think your teaching skills are not in play?
- What what would it take for you to use them more actively?
- Is it something you would like to do?

**Experiences with teaching**

- Where were you employed?
- How did you use your teaching skills?
- Who did you teach and how?
- How did it come about?
- How was it received? Examples of positive and negative experiences?
- Is there a difference between teaching students and colleagues - if yes, what?
- Would you say communication is considered important in your department?
- Is it taught? Formally/ad hoc?
- What would it take for you to use your CS skills more actively than you do now?
